# Exploring the relationship of supernumerary recurrent renal calculi formation and tick-borne infections: a case report

**DOI:** 10.3389/fcimb.2024.1194307

**Published:** 2024-01-26

**Authors:** Dean C. Paz, Abigael C. Gunther, Michael C. Higham, Lynne G. Stephenson, Anthony J. Laporta, K. Dean Gubler, Rebecca J. Ryznar

**Affiliations:** ^1^ Rocky Vista University College of Osteopathic Medicine, Parker, CO, United States; ^2^ Rocky Vista University College of Osteopathic Medicine, Ivins, UT, United States; ^3^ Department of Military Medicine, Rocky Vista University College of Osteopathic Medicine, Parker, CO, United States; ^4^ Department of Military Medicine, Rocky Vista University College of Osteopathic Medicine, Ivins, UT, United States; ^5^ Department of Biomedical Sciences, Rocky Vista University College of Osteopathic Medicine, Parker, CO, United States

**Keywords:** Cacchi-Ricci disease, medullary sponge kidney, nephrolithiasis, renal calculi, tick-borne infections, *Bartonella*, *Babesia*, *Borrelia*

## Abstract

A 51-year-old male with a history of Cacchi-Ricci disease and long-standing infection with various species of *Borrelia, Babesia, and Bartonella* presented with recurrent symptoms of right-sided flank pain. Numerous renal calculi were identified on imaging. The etiology of the calculi had not been previously elucidated. Symptoms intermittently date back to 2002 when uric acid stones were identified. Subsequent calculi analysis revealed calcium oxalate stones. Despite the commonality of nephrolithiasis in patients with Cacchi-Ricci disease, the extreme number of calculi and recurrent presentation of symptoms persisted despite a plethora of medical evaluations, dietary changes, and hereditary testing. This case raises questions of etiology including possible immune deficiency and whether his uncommon microbial history contributes to recurrent stone formation.

## Introduction

Cacchi-Ricci Disease, otherwise known as Medullary Sponge Kidney (MSK), is a congenital abnormality of the terminal collecting ducts commonly associated with recurrent nephrolithiasis (Gambaro et al., 2006). Nephrolithiasis is the production of urinary calculi within the kidney and often presents in symptomatic patients with renal colic and hematuria but may be asymptomatic in others depending on calculi size (Li et al., 2019). Commonly, renal calculi consist of calcium oxalate stones that arise due to elevated calcium or high oxalate concentrations in the urine. Other types of renal calculi include struvite, uric acid, calcium-phosphate, and cystine stones (Ratkalkar and Kleinman, 2011; Lieske et al., 2014). Risk factors for stone formation include dehydration, diets high in animal protein, electrolyte, vitamin, and mineral imbalances, medications, and obesity (Taylor et al., 2004; Atan et al., 2005; Taylor et al., 2005; Taylor et al., 2009).

Infections with *Borrelia*, *Babesia*, and *Bartonella* species have been linked to tick bites (Eskow et al., 2001; Vannier et al., 2015). *Borrelia* and *Babesia* infections are transmitted by the *Ixodes scapularis* tick while *Bartonella* infection is associated with bites from the *Ixodes ricinus* tick (Eskow et al., 2001; Steere et al., 2003). While not commonly thought of as a disease of the kidney, Lyme Disease, caused by *Borrelia burgdorferi*, has been linked to renal disease and stone formation (Ciută et al., 2012). In addition, concomitant *Bartonella* and *Babesia* infections have been hypothesized to result in increased disease severity (Knapp and Rice, 2015).

This case report explores a possible relationship between a microbial history with *Borrelia*, *Babesia*, and *Bartonella* species and supernumerary recurrent kidney stone formation in an individual with Cacchi-Ricci Disease. Evidence of the association between microbial pathogens and kidney stone formation has been inconclusive in previous studies. The abundance of stones seen in the patient presented in this report is confounding. Further investigation regarding the possible association is warranted.

## Case

A 51-year-old male with a history of Cacchi-Ricci disease and long-standing *B. Burgdorferi* Osp A (Outer Surface Lipoprotein A), *B Burgdorferi* Osp B-NPS (Outer Surface Lipoprotein B), *Babesia microti*, *Babesia Duncani*, *Bartonella henselae*-NPS, *Bartonella quintana*-NPS, *Borrelia miyamotoi*-NPS, *Borrelia recurrentis* sought medical care for recurrent symptoms of right-sided flank pain. Past medical history includes a tick bite at 8 years old while camping in Bear Lake, Utah. He was prescribed Tetracyclines for four years to treat acne while in high school.

**Figure 1:**
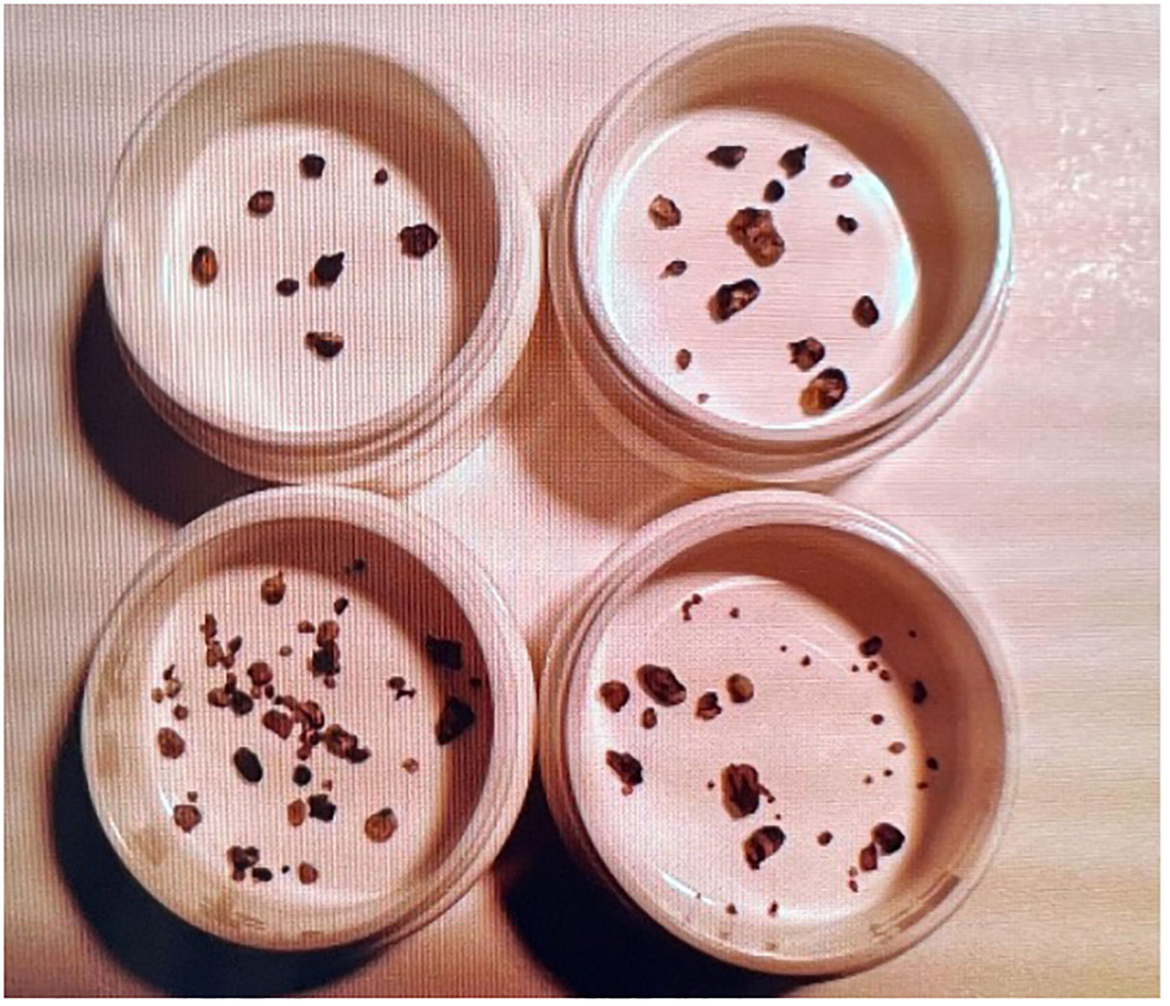
Renal calculi throughout the years. Microscopic image showcasing the assortment of renal calculi collected over several years. The top 2 containers are prior to 2021. The bottom left container is from 2021, and the bottom right corner is from 2022. The image highlights a multitude of supernumerary renal calculi, elucidating their recurrent formation. The correlation between these calculi and tick-borne infections is explored in this case report, presenting a potential link worthy of further investigation.

**Figure 2:**
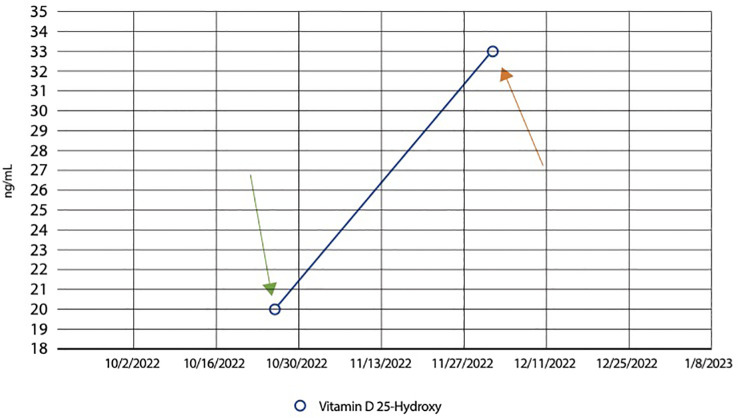
Line graph showing an increase in Vitamin D levels following supplementation. Graphical representation illustrating the patient’s dynamic Vitamin D levels over time. The patient initially had a level of 20ng/mL (normal range between 20 to 40ng/mL) on October 28^th^ 2022, as indicated by the green arrow. The graph demonstrates a significant rise in Vitamin D levels following supplementation. From an initial deficient state, the patient’s Vitamin D levels increased to 33 ng/mL, as seen by the orange arrow, showcasing the impact of Vitamin D supplementation. This observation is pertinent to the investigation into the relationship between supernumerary recurrent renal calculi formation and potential associations with tick-borne infections, offering insights into the potential role of Vitamin D in this context.

**Figure 3:**
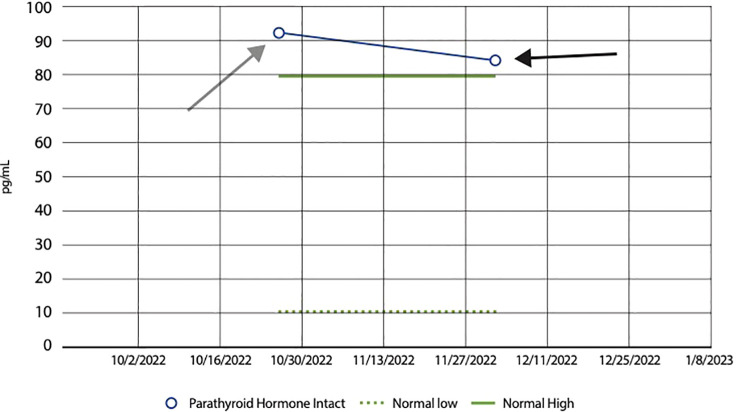
Line graph showing a decrease in PTH following Vitamin D supplementation. Bar graph showing the impact of Vitamin D supplementation on Parathyroid Hormone (PTH) levels in our patient. The graph displays a noticeable decrease from an initial PTH level of 92 pg/mL, as seen by the grey arrow, to 83 pg/mL, as shown by the black arrow, following Vitamin D supplementation. This decline in PTH levels suggests a regulatory effect of Vitamin D on parathyroid function; Vitamin D plays a pivotal role in calcium homeostasis. When Vitamin D levels are low, there is a resultant rise in PTH secretion from the parathyroid glands, aiming to increase calcium absorption. Consequently, when Vitamin D levels are high, PTH levels decrease. The normal range of PTH levels range from 10 to 80 pg/mL, as shown by the green dashed and green solid lines, respectively.

**Figure 4:**
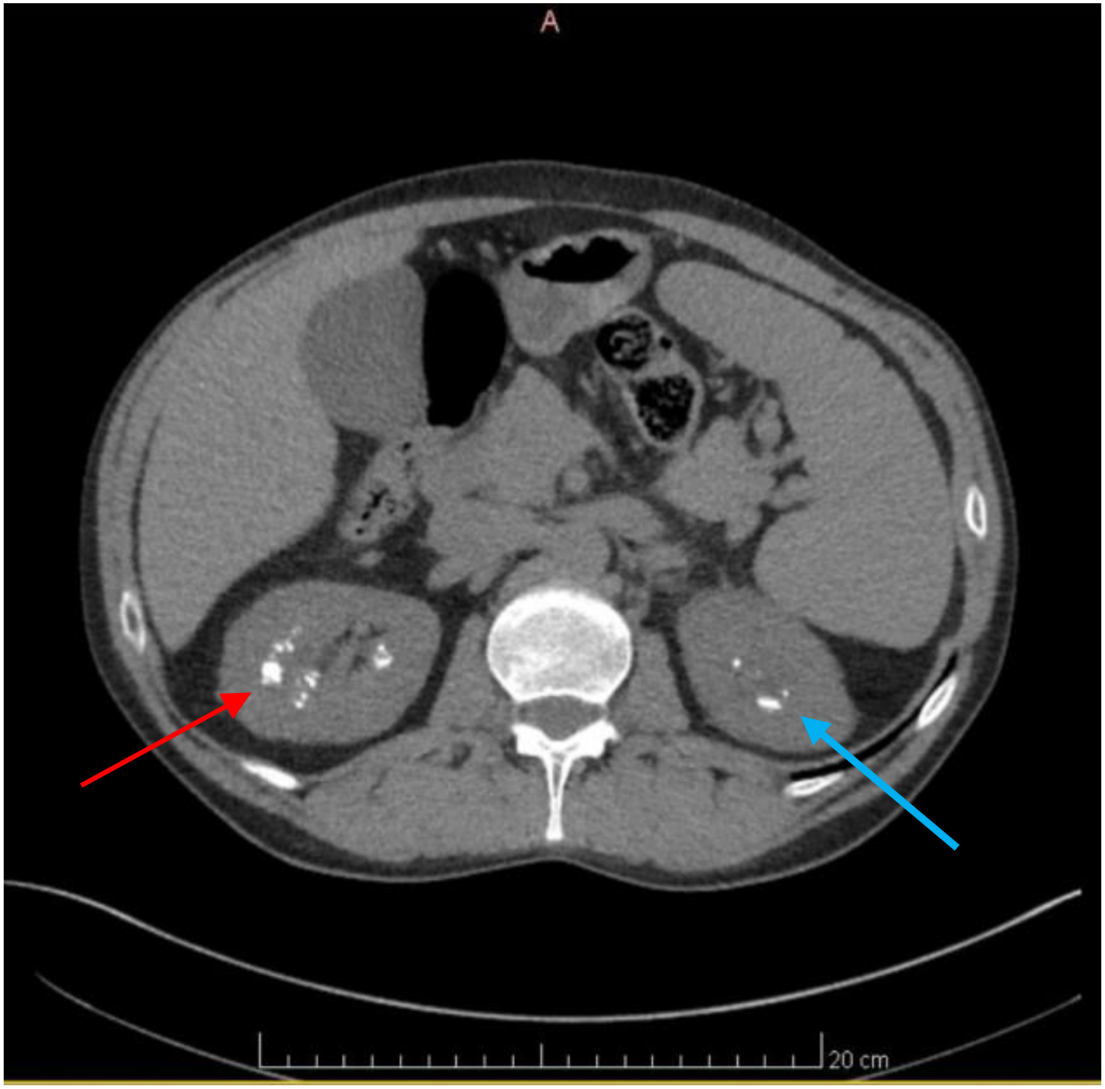
Axial CT scan of the Abdomen and Pelvis revealing numerous renal calculi. Axial CT scan images of the Abdomen and Pelvis depicting the presence of numerous renal calculi primarily localized in the right kidney, as seen by the red arrow. Additionally, visualization reveals the presence of renal calculi in the left kidney, albeit to a lesser extent, indicated by the blue arrow.

On presentation, he brought with him a renal calculi that he reportedly passed earlier in the year, ultimately hoping to understand the underlying etiology of the symptoms and history of nephrolithiasis. His history of renal lithiasis dates back to November 2002; the patient was 30-years-old at that time. Analysis of his recurrent stones in 2003 revealed uric acid stones; laboratory evaluation revealed an elevated serum uric acid level, 874 mg/dL. Allopurinol was prescribed with uric acid levels decreasing dramatically with an associated decrease in renal stone passage. Due to recurrent flank pain, radiological imaging was performed, which revealed generalized enlargement of the right kidney, blunted calyces and splenomegaly. A presumptive diagnosis was made of a possible variation of medullary sponge kidney. Symptoms persisted and increased in severity beginning again in 2017, despite medication use and dietary changes. The patient passed 57 stones in 2021 and 49 stones in 2022, as shown in **Figure 1**. On presentation to our urgent care, analysis of the stone he presented consisted of a Calcium Oxalate brown and tan stone that weighed 19 mg. Laboratory evaluation revealed deficient Vitamin D level of 20 ng/mL (nl range 30-100 ng/mL), and an elevated parathyroid hormone level of 92.3 pg/mL (nl range 10.8-79.4 pg/mL), **Figures 2**, **3**. These levels are ultimately consistent with the etiology of calcium oxalate crystal formation. Meanwhile, previous studies have revealed that Lyme disease is correlated with a Vitamin D deficiency (Donta, 2012). This may explain the reasoning behind his lab values, but further workup is required to make this conclusion. CT of the abdomen and pelvis revealed extensive nephrolithiasis in the right kidney, with sparse calculi in the left kidney, as seen in **Figure 4**.

On presentation, the patient reported being compliant with naturopathic medical management of his Vitamin D levels that consisted of Vitamin D supplements, mimosa pudica, and chanca Piedra. Despite changes in lifestyle, medication management, and herbal supplements, he passed two stones during the first 6 weeks of 2023. Herbal supplements included Mimosa Pudica, and Chanca Piedra, which were prescribed by a naturopath the patient has been seeing. At this point in time, the patient’s microbial conditions are being medically managed by an external primary care provider upon major dermatological flare-ups which include intermittent short-lasting pruritic rashes on his cubital fossa, forearm, and wrist.

## Discussion

Urolithiasis has been increasing in prevalence, and is a common source of renal injury in the aging population (Lang et al., 2022). The diagnosis of renal calculi is often clinical but can be confirmed with computerized imaging. As mentioned previously, there are different types of kidney stones, with calcium oxalate being the most common (Khan et al., 2016). The mechanism of stone formation is attributed to supersaturation in the urine but can also be due to dietary measures (Ratkalkar and Kleinman, 2011). Additional factors such as lifestyle, dietary factors, different kidney diseases, and metabolic syndromes also pose an elevated risk (Chung et al., 2013).

Medullary sponge kidney, originally known as Lenarduzzi-Cacchi-Ricci disease, is a congenital pathology characterized by the malformation of the pericalyceal terminal collecting ducts. Collecting duct dilatation is associated with formation of both small and large medullary “cysts” that rarely involve the cortex. The exact pathogenesis, and association with nephrocalcinosis and nephrolithiasis, is not fully understood and controversial (Rao et al., 1977; Ginalski et al., 1990; Yagisawa et al., 2001). One purported mechanism is believed to be a variant in the gene encoding glial cell-derived neurotrophic factor (GDNF), which is found to be down-regulated in MSK cells which is thought to promote calcium phosphate deposition. This combined with urine stagnation in dilated collecting tubules could precipitate stone formation (Mezzabotta et al., 2015). Additionally, metabolic abnormalities like idiopathic hypercalciuria prior to onset of hyperparathyroidism have been cited (Gambaro et al., 2006; Evan et al., 2015). Medullary sponge kidney patients are often asymptomatic, can present with recurrent urinary tract infections with or without nephrolithiasis. The diagnosis is usually made during investigation for hematuria or recurrent stone formations in conjunction with normal renal values (Garfield and Leslie, 2022). In this case, we present a patient with an abundance of renal calculi and historical vector-borne bacterial disease, underscoring the need to consider microbial infections as a possible source of urolithiasis.

Lyme disease is known as “the great masquerader” due to diffuse and vague symptomatology. The *Ixodes scapularis* tick is the vector responsible for Lyme disease (*B. burgdorferii*) and babesiosis (*B. microti*). Although the *Ixodes* tick transmits both diseases, coinfections are believed to be relatively rare, 2% (Steere et al., 2003). It is suggested that co-infection may result in a more prolonged and severe course of illness. Symptoms of chronic disease typically include fatigue, arthralgias/myalgias, and cognitive dysfunction. The role and pathogenesis of Lyme disease in nephrolithiasis has not been extensively detailed with only a few case reports documenting this unique connection (Ciută et al., 2012; Reeze and Maru, 2016). A possible explanation includes the invasive capabilities of the organism. Distinct dissemination features by *B. burgdorferi* include interactions between the p66 outer membrane protein and host target cell integrins that play a role in transendothelial migration. The kidney has extensive post-capillary venules that express β1 integrins, and although this may be a less common target of the p66 outer membrane protein, binding could potentiate a mechanism for hematogenous dissemination within host tissues (Hyde, 2017; Mohamed et al., 2020).

Infection secondary to *Babesia microti* is the primary source of babesiosis in humans in North America. Babesiosis is characterized by a gradual onset of fever, fatigue, and malaise accompanied by chills, sweats, headaches, and myalgia. It is transmitted by the *Ixodes scapularis* tick which is commonly found on mice and other small rodents (Smith et al., 2020; Waked and Krause, 2022). In humans, acute tubular injury has been reported with babesiosis secondary to volume depletion and heme pigment toxicity following hemolysis caused by the infection (Luciano et al., 2013). While there is not much more research on the impact of babesiosis on human kidneys, work has been done to identify its impact on animal models. In rats, *B. microti* has shown a chemotactic tendency to adhere to the epithelium of renal tubules. Involvement within the epithelial cells showed a decreased ability to fight against oxidative stress, an increase in free radicals, and resultant tissue damage and necrosis (Albertyńska et al., 2021). Further studies in mice showed *B. microti* infection leading to renal abnormalities including abnormal serum creatinine, uric acid, and bilirubin levels (Li et al., 2022). Considering that rodents are a natural reservoir for *B. microti*, further research is necessary to see how these results would translate to human kidneys, including the impact on nephrolithiasis formation.

Additionally*, Bartonella henselae*, commonly manifesting as Cat Scratch disease, can also cause a wide spectrum of clinical signs in association with persistent infection. There is a substantial reservoir in both domestic and wild animals that can result in accidental human infection via the *Ixodes ricinus* tick (Eskow et al., 2001). Although classically associated with cats via fleas, the knowledge of vector transmission for *Bartonella* remains to be delineated (Chomel et al., 1996). For protracted courses of Lyme’s disease refractory to initial treatment, it has been suggested that a second pathogen, such as *Bartonella*, might be the cause (Berghoff, 2012). The compounding effects of these bacteria on the kidney is only hypothesized at this point, however, one proposed mechanism of pathology is the formation of reactive oxygen species (Eskow et al., 2001; Khan, 2004). The normal urinary environment is not conducive to kidney stone crystallization. Reactive oxygen species that are produced during renal cell injury stimulate the production of products like osteopontin, prostaglandin E_2_, and heparan sulfate proteoglycan, which are known modulators of inflammation and production of extracellular matrix. Renal epithelial cells that are exposed to inflammatory mediators undergo a cycle of apoptosis and necrosis that lead to further crystallization and stone formation (Khan, 2013).

Although previous documentation of infection associated renal calculi have been inconclusive, the etiology behind the numerous stones our patient has experienced remains to be determined. His unique history of infection with multiple intracellular pathogens, however, cannot be excluded as contributory, and a history of vector borne infections should be considered in the clinical evaluation of urolithiasis.

## Conclusion

This case highlights the importance of a comprehensive approach towards evaluating and managing recurrent nephrolithiasis in patients with an extensive complex microbial history, particularly Lyme disease. It is crucial to consider both infectious and metabolic factors, as well as their potential interactions, when evaluating such patients. Continuous monitoring and management of chronic infections, combined with appropriate evaluation and treatment of metabolic abnormalities, are key to preventing further stone formation and managing symptoms. Further research to determine the potential correlation between the patient’s infectious medical history and recurrent nephrolithiasis is warranted. By considering both infectious and metabolic factors, healthcare providers can provide optimal care, prevent further stone formation, and improve patient outcomes.

## Data availability statement

The original contributions presented in the study are included in the article/supplementary material. Further inquiries can be directed to the corresponding author.

## Ethics statement

Written informed consent was obtained from the individual(s) for the publication of any potentially identifiable images or data included in this article. Written informed consent was obtained from the patient for the publication of this case report.

## Author contributions

DP, AG, and MH were responsible for conceptualization, data curation, formal analysis, investigation, methodology, resources, visualization of the work, writing of the original draft and reviewing. DP was also responsible for project administration and supervision of the project. DP prepared **Figures 1**–**4**, and KG reviewed them prior to submission. KG, RR, and AL were responsible for reviewing and editing the work, along with oversight of the execution of the project. LS was responsible for resource acquisition. All authors contributed to the article and approved the submitted version.

## References

[B1] Albertyńska M. Okła H. Jasik K. Urbańska-Jasik D. Pol P. (2021). Interactions between Babesia microti merozoites and rat kidney cells in a short-term *in vitro* culture and animal model. Sci. Rep. 11 (1), 23663. doi: 10.1038/s41598-021-03079-0 34880327 PMC8654915

[B2] Atan L. Andreoni C. Ortiz V. Silva E. K. Pitta R. Atan F. . (2005). High kidney stone risk in men working in steel industry at hot temperatures. Urology 65 (5), 858–861. doi: 10.1016/j.urology.2004.11.048 15882711

[B3] Berghoff W. (2012). Chronic lyme disease and co-infections: differential diagnosis. Open Neurol. J. 6, 158–178. doi: 10.2174/1874205X01206010158 23400696 PMC3565243

[B4] Chomel B. B. Kasten R. W. Floyd-Hawkins K. Chi B. Yamamoto K. Roberts-Wilson J. . (1996). Experimental transmission of Bartonella henselae by the cat flea. J. Clin. Microbiol. 34, 1952–1956. doi: 10.1128/jcm.34.8.1952-1956.1996 8818889 PMC229161

[B5] Chung C. Stern P. J. Dufton J. (2013). Urolithiasis presenting as right flank pain: a case report. J. Can. Chiropr Assoc. 57 (1), 69–75. 23483000 PMC3581005

[B6] Ciută C. Nechifor V. Tomac I. Miron A. Novac B. Novac C. (2012). Lyme disease–unusual medical encounter for an urologist. Rev. Med. Chir Soc. Med. Nat. Iasi 116 (4), 1101–1105. 23700896

[B7] Donta S. T. (2012). Issues in the diagnosis and treatment of lyme disease. Open Neurol. J. 6, 140–145. doi: 10.2174/1874205X01206010140 23248715 PMC3520031

[B8] Eskow E. Rao R. V. Mordechai E. (2001). Concurrent infection of the central nervous system by Borrelia burgdorferi and Bartonella henselae: evidence for a novel tick-borne disease complex. Arch. Neurol. 58 (9), 1357–1363. doi: 10.1001/archneur.58.9.1357 11559306

[B9] Evan A. P. Worcester E. M. Williams J. C. Jr Sommer A. J. Lingeman J. E. Phillips C. L. . (2015). Biopsy proven medullary sponge kidney: clinical findings, histopathology, and role of osteogenesis in stone and plaque formation. Anat. Rec. (Hoboken) 298 (5), 865–877. doi: 10.1002/ar.23105 25615853 PMC4405475

[B10] Gambaro G. Feltrin G. P. Lupo A. Bonfante L. D’Angelo A. Antonello A. (2006). Medullary sponge kidney (Lenarduzzi-Cacchi-Ricci disease): a Padua Medical School discovery in the 1930s. Kidney Int. 69 (4), 663–670. doi: 10.1038/sj.ki.5000035 16395272

[B11] Garfield K. Leslie S. W. (2022). “Medullary sponge Kidney,” in StatPearls (Treasure Island (FL: StatPearls Publishing). Available at: https://www.ncbi.nlm.nih.gov/books/NBK470220/. 29262095

[B12] Ginalski J. M. Portmann L. Jaeger P. (1990). Does medullary sponge kidney cause nephrolithiasis? AJR Am. J. Roentgenol. 155 (2), 299–302. doi: 10.2214/ajr.155.2.2115256 2115256

[B13] Hyde J. A. (2017). Borrelia burgdorferi keeps moving and carries on: A review of borrelial dissemination and invasion. Front. Immunol. 8. doi: 10.3389/fimmu.2017.00114 PMC5318424 28270812

[B14] Khan S. R. (2004). Role of renal epithelial cells in the initiation of calcium oxalate stones. Nephron. Exp. Nephrol. 98 (2), e55–e60. doi: 10.1159/000080257 15499208

[B15] Khan S. R. (2013). Reactive oxygen species as the molecular modulators of calcium oxalate kidney stone formation: evidence from clinical and experimental investigations. J. Urol. 189 (3), 803–811. doi: 10.1016/j.juro.2012.05.078 23022011 PMC5683176

[B16] Khan S. R. Pearle M. S. Robertson W. G. Gambaro G. Canales B. K. Doizi S. . (2016). Kidney stones. Nat. Rev. Dis. Primers 2, 16008. doi: 10.1038/nrdp.2016.8 27188687 PMC5685519

[B17] Knapp K. L. Rice N. A. (2015). Human Coinfection with Borrelia burgdorferi and Babesia microti in the United States. J. Parasitol. Res. 2015, 587131. doi: 10.1155/2015/587131 26697208 PMC4677215

[B18] Lang J. Narendrula A. El-Zawahry A. Sindhwani P. Ekwenna O. (2022). Global trends in incidence and burden of urolithiasis from 1990 to 2019: an analysis of global burden of disease study data. Eur. Urol. Open Sci. 35, 37–46. doi: 10.1016/j.euros.2021.10.008 35024630 PMC8738898

[B19] Li M. Yang X. Masoudi A. Xiao Q. Li N. Wang N. . (2022). The regulatory strategy of proteins in the mouse kidney during Babesia microti infection. Exp. Parasitol. 235, 108232. doi: 10.1016/j.exppara.2022.108232 35227683

[B20] Li X. Zhu W. Lam W. Yue Y. Duan H. Zeng G. (2019). Outcomes of long-term follow-up of asymptomatic renal stones and prediction of stone-related events. BJU Int. 123 (3), 485–492. doi: 10.1111/bju.14565 30253029

[B21] Lieske J. C. Rule A. D. Krambeck A. E. Williams J. C. Bergstralh E. J. Mehta R. A. . (2014). Stone composition as a function of age and sex. Clin. J. Am. Soc. Nephrol. 9 (12), 2141–2146. doi: 10.2215/CJN.05660614 25278549 PMC4255407

[B22] Luciano R. L. Moeckel G. Palmer M. Perazella M. A. (2013). Babesiosis-induced acute kidney injury with prominent urinary macrophages. Am. J. Kidney Dis. 62 (4), 801–805. doi: 10.1053/j.ajkd.2013.02.376 23643302

[B23] Mezzabotta F. Cristofaro R. Ceol M. Del Prete D. Priante G. Familiari A. . (2015). Spontaneous calcification process in primary renal cells from a medullary sponge kidney patient harbouring a GDNF mutation. J. Cell Mol. Med. 19 (4), 889–902. doi: 10.1111/jcmm.12514 25692823 PMC4395202

[B24] Mohamed T. H. Watanabe H. Kaur R. Belyea B. C. Walker P. D. Gomez R. A. . (2020). Renin-expressing cells require β1-integrin for survival and for development and maintenance of the renal vasculature. Hypertension 76 (2), 458–467. doi: 10.1161/HYPERTENSIONAHA.120.14959 32594804 PMC7347440

[B25] Rao D. S. Frame B. Block M. A. Parfitt A. M. (1977). Primary hyperparathyroidism: A cause of hypercalciuria and renal stones in patients with medullary sponge kidney. JAMA 237 (13), 1353–1355. doi: 10.1001/jama.1977.03270400057021 576483

[B26] Ratkalkar V. N. Kleinman J. G. (2011). Mechanisms of stone formation. Clin. Rev. Bone mineral Metab. 9 (3-4), 187–197. doi: 10.1007/s12018-011-9104-8 PMC3252394 22229020

[B27] Reeze Z. Maru P. (2016). Unexplained recurrent fevers and the importance of inquiring about occupation: A Case Report. TMF 17 (1), 11. doi: 10.29046/TMF.017.1.012

[B28] Smith R. P. Hunfeld K. P. Krause P. J. (2020). Management strategies for human babesiosis. Expert Rev. Anti Infect. Ther. 18, 625–636. doi: 10.1080/14787210.2020.1752193 32268823

[B29] Steere A. C. McHugh G. Suarez C. Hoitt J. Damle N. Sikand V. K. (2003). Prospective study of coinfection in patients with erythema migrans. Clin. Infect. Dis. 36, 1078–1081. doi: 10.1086/368187 12684924

[B30] Taylor E. N. Fung T. T. Curhan G. C. (2009). DASH-style diet associates with reduced risk for kidney stones. J. Am. Soc. Nephrol. 20 (10), 2253–2259. doi: 10.1681/ASN.2009030276 19679672 PMC2754098

[B31] Taylor E. N. Stampfer M. J. Curhan G. C. (2004). Dietary factors and the risk of incident kidney stones in men: new insights after 14 years of follow-up. J. Am. Soc. Nephrol. 15 (12), 3225–3232. doi: 10.1097/01.ASN.0000146012.44570.20 15579526

[B32] Taylor E. N. Stampfer M. J. Curhan G. C. (2005). Obesity, weight gain, and the risk of kidney stones. JAMA 293 (4), 455–462. doi: 10.1001/jama.293.4.455 15671430

[B33] Vannier E. G. Diuk-Wasser M. A. Ben Mamoun C. Krause P. J. (2015). Babesiosis. Infect. Dis. Clin. North Am. 29 (2), 357–370. doi: 10.1016/j.idc.2015.02.008 25999229 PMC4458703

[B34] Waked R. Krause P. J. (2022). Human babesiosis. Infect. Dis. Clin. North Am. 36 (3), 655–670. doi: 10.1016/j.idc.2022.02.009 36116841

[B35] Yagisawa T. Kobayashi C. Hayashi T. Yoshida A. Toma H. (2001). Contributory metabolic factors in the development of nephrolithiasis in patients with medullary sponge kidney. Am. J. Kidney Dis. 37 (6), 1140–1143. doi: 10.1053/ajkd.2001.24515 11382681

